# Micro(nano)plastics and Their Potential Impact on Human Gut Health: A Narrative Review

**DOI:** 10.3390/cimb46030168

**Published:** 2024-03-21

**Authors:** Carlo Covello, Federica Di Vincenzo, Giovanni Cammarota, Marco Pizzoferrato

**Affiliations:** 1 Center for Diagnosis and Treatment of Digestive Diseases, Gastroenterology Department, Fondazione Policlinico Universitario Agostino Gemelli IRCCS, 00168 Rome, Italy; covellocarlo@gmail.com (C.C.); federica.divincenzo30@gmail.com (F.D.V.); 2 UOC Gastroenterologia, Dipartimento di Scienze Mediche e Chirurgiche, Fondazione Policlinico Universitario Agostino Gemelli IRCCS, 00168 Rome, Italy; giovanni.cammarota@unicatt.it

**Keywords:** digestive system, gut microbiota, human cells, intestinal toxicity, microplastics, mouse models, nanoplastics, plastic, polymers

## Abstract

Microplastics and nanoplastics (MNPs) are becoming an increasingly severe global problem due to their widespread distribution and complex impact on living organisms. Apart from their environmental impact, the effects of MNPs on living organisms have also continued to attract attention. The harmful impact of MNPs has been extensively documented in marine invertebrates and larger marine vertebrates like fish. However, the research on the toxicity of these particles on mammals is still limited, and their possible effects on humans are poorly understood. Considering that MNPs are commonly found in food or food packaging, humans are primarily exposed to them through ingestion. It would be valuable to investigate the potential harmful effects of these particles on gut health. This review focuses on recent research exploring the toxicological impacts of micro- and nanoplastics on the gut, as observed in human cell lines and mammalian models. Available data from various studies indicate that the accumulation of MNPs in mammalian models and human cells may result in adverse consequences, in terms of epithelial toxicity, immune toxicity, and the disruption of the gut microbiota. The paper also discusses the current research limitations and prospects in this field, aiming to provide a scientific basis and reference for further studies on the toxic mechanisms of micro- and nanoplastics.

## 1. Introduction

Microplastics (MPs) are tiny particles derived from plastics, or synthetic or semisynthetic polymers produced from hydrocarbon or biomass materials. Most plastics are petroleum-derived polymers that consist of “molecules of high relative molecular mass, whose structure essentially comprises multiple repetitions of derived units, from molecules of low relative molecular mass” [1]. These polymers, like polypropylene (PP), polyethylene (PE), polyvinyl chloride (PVC), polyethylene terephthalate (PET), and polystyrene (PS), are non-biodegradable [2].

Plastic is a widely used material in industrial applications and its production has been consistently increasing over the years. In 2016, annual plastic production reached 300 million tons. However, if this trend continues, it is estimated that approximately 25 million tons of plastic waste will be produced by 2050 [3,4,5,6,7]. Plastic is a key component in a diverse range of industrial and consumer products, including cosmetics, detergents, paints, synthetic fertilizers, and pesticides, among others. Unfortunately, MPs have been detected in various food products, such as processed foods, beer, seafood, and sugar-sweetened beverages [8,9].

This widespread plastic contamination, which has been attributed to the limited recycling efforts and the absence of regulatory frameworks, has had a substantial impact on aquatic, terrestrial, and atmospheric environments. The issue of plastic pollution has become a pressing concern as it is now present in almost all water bodies, including oceans, seas, rivers, and lakes, thereby posing a significant threat to biodiversity and public health [10].

Plastic waste, once discarded in nature, is exposed to different factors, including physical (e.g., ultraviolet radiation and temperature), chemical (e.g., salinity, pH, and corrosive agents), and biological (e.g., bacteria, microalgae, and plankton). These factors decompose plastic waste into particles of different sizes and ecological impact. Three main classification groups are commonly used to describe plastic waste based on its particle size: macro- (>25 mm), meso- (between 5 and 25 mm), and microplastics (MPs < 5 mm). Additionally, the intentional production and further degradation of microplastics can generate smaller waste particles, known as nanoplastics (NPs < 1 µm) [11].

Microplastics are categorized by their origin. “Primary” microplastics are intentionally created at the microscale, while “secondary” microplastics come from the fragmentation of larger plastics [12].

Microplastic pollution is widespread in soil environments, including agricultural soils, greenhouses, and coastal, industrial, and floodplain soils. This type of pollution is a result of the inappropriate management and unsustainable use of plastic waste and agricultural processes [13,14]. Microplastic pollution is also a significant issue in aquatic environments, such as the marine environment, where plastic debris can be found on the sea floor, surface, and shoreline [15]. It has been estimated that 80% of the plastic pollution in oceans and seas comes from land [16]. Microplastics have also been detected in freshwater, including lakes, rivers, and groundwater. These particles mainly come from urban pollution, shipping, fishing, tourism, oil and gas platforms, wastewater treatment plants, discharged personal care products, textiles, and packaging [17]. Furthermore, microplastics have been found in the atmospheric fallout in both megacities and sparsely populated areas [6,18,19,20,21], and suspended atmospheric microplastics have also been repeatedly detected in indoor air [22,23].

The wide distribution of microplastics and nanoplastics (MNPs) promotes contamination by different animal species, especially by integumentary exposure, inhalation, and ingestion [24,25,26]. Specifically, for mammals and humans, the inhalation of nanomaterials and ingestion of contaminated water, sea salt, and seafood are the main routes of exposure to these plastic particles [27].

Although previously considered safe and inert materials, the negative biological impact of the contamination of microplastics and nanoplastics has been demonstrated recently [26,28,29]. As a result, the smallest particles (such as 10 μm and 2.5 μm) can penetrate organs like lungs and intestines, as well as cells like enterocytes and macrophages. These particles are recognized as foreign elements that stimulate immune response and oxidative stress [29,30]. Due to their difficulty in being cleared biologically, particles can accumulate and result in chronic inflammation, potentially leading to the development of tumors [29,31,32]. In addition, microplastics and nanoplastics pose a high toxicological risk, as they contain hazardous additives like plasticizers, flame retardants, stabilizers, dyes, antistatic agents, lubricants, sliding agents, curing agents, foaming agents, and biocides [33]. It is worth noting that microplastics have the potential to adopt a fibrous form, which is commonly referred to as “microplastic fibers” [34]. The contamination of environments with microplastic fibers is probably as much as, or even more than, that caused by microplastic particles [35,36]. Because of their elongated shape, microplastic fibers have a higher potential for bioaccumulation and can cause direct harm to organisms or lead to adverse effects [37].

It is currently not feasible to conduct clinical studies that analyze the health risks of MNPs in humans due to ethical concerns. As a result, we do not have a clear understanding of the health impact of MNPs on humans. We are unaware of the extent to which humans can absorb and accumulate MNPs, and the pharmacokinetic and pharmacodynamic mechanisms associated with them [38].

However, it is still a major concern that MNPs could have toxicological effects on the entire intestinal system, especially through ingestion, which remains one of the main exposure mechanisms of these particles.

This paper aims to present an objective overview of the potential impacts that these polymers could have on the intestinal system by highlighting the toxicological effects related to MNPs for in vivo mammalian and in vitro human cell studies found in the literature. This review focuses specifically on the toxicological effects of MNPs on the gut and the complex immunological system related to it, examining the various interrelationships that these particles have with the gut microbiota. These studies cannot provide clinical data. However, they can lay an important foundation for future research by providing an overview of these issues.

## 2. Main Pathogenetic Mechanisms of MNP-Induced Cell Toxicity

Experimental models have revealed that the mechanisms of membrane damage, oxidative stress, immune response, and genotoxicity contribute to the toxicity of MNPs.

Among them, the cytotoxicity of MNPs was mainly attributed to membrane damage and oxidative stress [39]. Particles can damage the plasma membrane, which is often observed with cationic particles [40,41]. Polyethylene nanoparticles have been found to penetrate the hydrophobic milieu of the bilayer of the plasma membrane and cause structural changes [42]. Endocytosed particles can permeabilize the endosomal and lysosomal membrane and interact with intracellular organelles [43,44].

Reactive oxygen species (ROS) can be generated during plastic polymerization and particle processing and, upon interaction with the bioenvironment, cause cellular stress [45]. On the other hand, the direct or indirect impairment of DNA through the translocation of particles or ROS into the nucleus and damage to the DNA replication or repair mechanism may contribute to the genotoxicity of particles [46,47].

In mammalian cells, MNPs can cause nuclear membrane disruption, oxidative stress, the release of damage-associated molecular patterns, and the downstream activation of inflammatory and apoptotic and necrotic pathways [44,48].

The absorption of micro- or nanoplastics can lead to the loss of integrity of plasma, endosomal, and nuclear membranes, causing pore formation in membranes and the subsequent generation of ROS from mitochondria. Elevated levels of intracellular ROS can cause mitochondrial damage due to increased mitochondrial Ca2+, concomitant mitochondrial membrane depolarization, the release of pro-apoptotic factors from mitochondria, the reduction of ATP, the release of damage-associated molecular patterns (DAMPs) from mitochondria or other organelles, resulting in the production of pro-inflammatory cytokines, and, finally, the activation of cell death pathways, leading to apoptosis or necrosis [38].

## 3. MNPs and the Intestinal System

The main source of exposure to microplastic and nanoplastic particles is through the ingestion of food or water that is contaminated with these particles. Plastics inevitably find their way into the food chain and carry contaminants that can affect intestinal homeostasis. Studies have found the presence of microplastics and nanoplastics in many types of foods, including fruits, vegetables, marine products, livestock (such as chickens), and drinking water [49,50,51,52,53]. Other foods such as sugar, honey, beer, cow’s milk, and sea salt have also been found to contain microplastics [9,52,54,55,56]. These particles have even been found in the gastrointestinal contents of more than 220 different marine species, such as *mussels*, *oysters*, *clams*, and *common shrimp*, as well as in various seafood products [57,58,59].

The most commonly detected polymers in food and drinking water are polyethylene (PE), polypropylene (PP), polystyrene (PS), polyvinyl chloride (PVC), and polyethylene terephthalate (PET). Polyamide (PA), acrylic, or acrylic-related compounds, polyesters, and polymethyl methacrylate (PMMA) are also detected, but less frequently [60].

It is uncertain whether the ingestion of MNPs poses a significant risk to the intestinal system, given the conflicting data on human exposure and the biodistribution of these particles. It has been observed that human adults can potentially ingest up to 458,000 microplastic particles per annum through tap water and 3,569,000 microplastic particles per annum through bottled water [61]. However, there exists a considerable variation in the estimates of human exposure to microplastics due to differences in the type of plastic and experimental methodologies employed in various studies [9]. In a recent study, Schawabl et al. endeavored to estimate human contamination by measuring the amount of microplastics in the feces of eight healthy volunteers. The study established an average of 20 microplastic particles per 10 g of feces, ranging in size from 50 to 500 μm, and belonging to nine types of plastics, with PP and PET being the most prevalent [62,63].

The distribution of micro- and nanoplastics after ingestion is not well-understood. Due to the stability of plastic materials, enzymatic or chemical degradation is challenging, especially since mammalian intestines lack specialized enzymes for plastic degradation. This means that plastic particles are not significantly degraded during digestion. Larger microplastics (>150 μm) remain attached to the intestinal mucus layer, directly contacting the apical part of intestinal epithelial cells. Smaller particles (<150 μm), however, can cross the intestinal mucus layer. The uptake of micro- and nanoplastics depends on their size and occurs through various mechanisms, including transcytosis through microfold cells, endocytosis through enterocytes, persorption (which is the passage through crevices at the end of the villus, following the loss of enterocytes), and paracellular uptake [64,65,66,67].

When micro- and nanoplastics are ingested, most of them are excreted through feces [68,69], while a small portion stays in the intestine for several days [68]. In the gut, MNPs can cause damage and inflammation by entering the bloodstream, spreading to other tissues, and persisting for prolonged periods [70]. The bioavailability of MNPs after oral intake depends on intestinal translocation. In a study of three intestinal cell models [71], it was observed that the size and surface chemistry of the particles influenced translocation, with 50 nm nanoparticles having a higher translocation rate than 100 nm NPs. The translocation of MNPs is influenced by various factors, including the characteristics of plastic particles and animal behavior and development [72].

Despite the low rate of intestinal absorption, exposure to significant amounts of micro- and nanoparticles could lead to systemic toxicity, as their small size allows them to penetrate deep into organs. Specifically, a study by Walczak et al. investigating the impact of in vitro gastrointestinal digestion on the protein crown of PS-NPs revealed that, after digestion, translocation was 4 times higher for positively charged NPs and 80 and 1.7 times higher for two different types of negatively charged NPs. In vitro digestion also reduced the presence of higher-molecular-weight proteins, shifting the protein content of the corona toward lower-molecular-weight proteins [73].

Comprehensive studies of the 55 most widely used polymer types developed a model for ranking the hazard of each polymer, according to the monomer chemicals that formed the polymer. The most hazardous polymers were those produced from carcinogenic, mutagenic, or both monomers. Hazard classification data were mainly taken from Annex VI of the EU Classification, Labelling and Packaging (CLP) regulation which is based on the UN Globally Harmonized System (GHS). However, while this approach determined a high ranking for polyurethanes, epoxy resins, and polyvinylchloride and styrene polymers, no hazard classification was available for many of the listed substances, such as suspected endocrine disruptors, due to the lack of safety data [67,74].

### 3.1. Toxicity of Micro-/Nanoplastics in the Intestine

**Figure 1:**
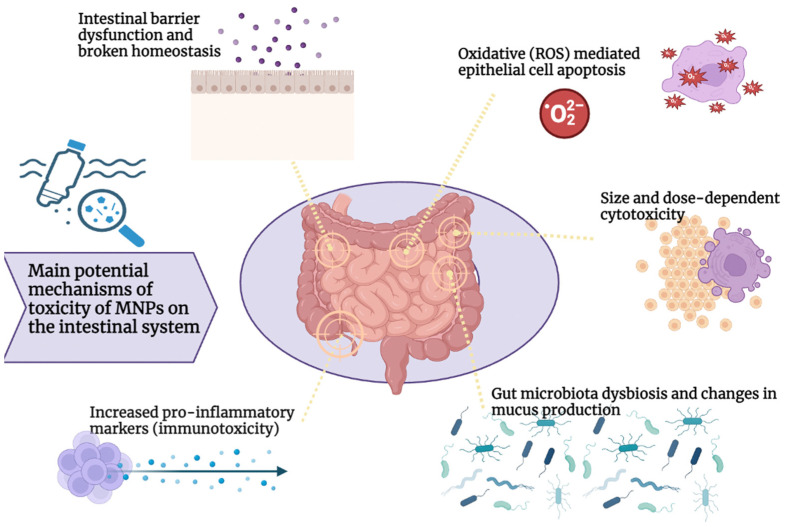
Main potential mechanisms of MNP toxicity on the intestinal system.

The effects of MNPs on the intestinal system and gut microbiota in mammals and humans, and the associated mechanisms, are still not fully understood. Figure 1 summarizes the main postulated toxicological effects of MNPs on the intestinal system.

Studies have shown that microparticles have a harmful impact on the intestines of invertebrates and vertebrates like fish. For instance, research conducted on *Caenorhabditis elegans*, *Artemia parthenogenetica* zooplankton larvae, and *Eisenia fetida* earthworm has revealed that intestinal oxidative damage is a significant mechanism in microplastic toxicity. Moreover, exposure to microparticles was found to be associated with the progression of cellular deformations and enterocyte decomposition [75,76,77]. Further evidence comes from studies that involved oral exposure to microplastics in aquatic vertebrates such as *rainbow trout*, *juvenile intertidal fish Girella laevifrons*, *juvenile large yellow croaker Larimichthys crocea*, or *Oryzias melastigma*. Overall, these studies have found that PS microbeads and nanoparticles cause a decrease in digestive enzyme activity (lipase, trypsin, and lysozyme) [78,79], and induce goblet cell enlargement and increased mucus secretion [78,80], the secretion of proinflammatory cytokines like Tumor Necrosis Factor α (TNF α), Interferon γ (IFN γ), and Interleukin-6 (IL-6) [78], leukocyte infiltration, hyperemia, and the loss of villi and crypt cells [81]. Interestingly, intestinal levels of oxidative stress enzymes were found to be modified in opposite ways between nano- and microparticles [80].

The toxic effects of microplastics on the gut have been assessed in several aquatic species, pointing out inflammation, genotoxicity, and oxidative stress responses [82]. Several studies have been conducted to investigate the effects of PS on the intestines of zebrafish. Exposure to PS beads resulted in an increase in the secretion of proinflammatory cytokines such as Interleukin-1α (IL1α), Interleukin-1β (IL1β), and IFN γ. This exposure also enhanced the activity of enzymes that respond to excessive oxidative stress. It was observed that exposure to PS was associated with reduced levels of antioxidant enzyme diamine oxidase and of D-lactate, which could indicate increased intestinal permeability [83]. Furthermore, a single-cell analysis revealed a dysfunction of intestinal cell populations, a decrease in the detoxification/antioxidant capacity of enterocytes, and a decrease in the cell chemotaxis of secretory cells.

It appears that the impact of microplastics on the intestinal epithelium depends not only on the size of the particles but also on their shape. In fact, when exposed to microplastic fibers, the volume of mucus in the intestine of zebrafish declined sharply. Additionally, both microplastic fibers and fragments led to a decrease in intestinal D-lactate, caused inflammation in the intestine, and increased the activity of superoxide dismutase [84]. Exposure to PVC induced a histological alteration in the intestine of European sea bass *Dicentrarchus labrax* L. [85], increasing the globet cell number, villus thickness, and expression of intestinal nuclear factor E2-related factor 2 (Nrf2). On the other side, exposure to irregularly shaped high-density PE and PS particles determined an epithelial detachment, increase in the neutrophil count, and decrease in the globet cell count in the intestine of zebrafish [86].

Notwithstanding the available evidence, the data from in vitro and in vivo studies in mammalian models are comparatively restricted and conflicting (Table 1).

In 2018, Abdelkhaliq et al. showed no cytotoxicity of polystyrene (PS) particles (50 nm and 200 nm) on Caco-2 cells at a concentration of 250 mg/mL for 10 to 120 min of exposure [87]. Accordingly, with 1 to 30 mg/mL, laser-ablated, approximately 100 nm PET particles, no impact on Caco-2 cell viability and no inflammation was measured up to 24 h of incubation [88]. Similarly, Hesler et al., in 2019, showed the absence of toxicity at a concentration below 100 mg/mL PS particles (between 40–52 nm and 457–477 nm) after 24 h of incubation [89]. A significant decrease of Caco-2 cell viability was only measured at very high concentrations of 4–10 µm PS particles (1 × 10^8^ particles per mL) after 48 h of incubation. Furthermore, in investigating the effect of PS particles on the macrophage cell line THP-1, no effect on cell polarization was detected after particle exposure [91]. According to a recent study, when HRT-18 and CMT-93 epithelial cell lines were exposed to PS microparticles (with a diameter of 4.8–5.8 µm, a concentration of 1 mg/mL, and a time between 6 and 48 h), it resulted in a significant increase in cytotoxicity in both cell lines. However, only CMT-93 cells showed an increase in oxidative stress activity [92]. Moreover, after being tested at various concentrations for 48 h, polyethylene (PE) microplastics between 30 and 140 μm caused a significant reduction in Caco-2 cell viability at high concentrations (1000 mg/L) [90].

Notably, a recent comparative systematic analysis monitored the influence of small microplastics, of size 50–100 nm, on human colon cells and human colon organoids, and in vivo in a mouse model. According to the authors, the viability of colon organoids decreased by over 20% when exposed to concentrations of 5 mg/mL of MPs. This exposure also led to an increase in the expression of genes linked to inflammation, apoptosis, and immunity. Additionally, in vivo data from a murine model indicated that 50 nm MPs accumulated in several mouse organs, including the colon, after 7 days of exposure [98].

Several studies in mice exposed to PS microspheres have shown a transcriptional decrease in major genes related to mucin expression, such as mucin 1 (Muc1) and Klf4 [93,94], and to ion transport, such as cystic fibrosis transmembrane conductance regulator (Cftr), Na-K-2Cl cotransporter 1 (Nkcc1), Na+/H+ exchanger 3 (Nhe3), anoctamin 1 (Ano1), and solute carrier family 26 member 6 (Slc26a6) [93]. In a research study conducted on mouse models, it was found that exposure to a mixture of microplastics ranging from 1 µm to 10 µm in size, at a volume of 10 mL/kg, and for a total of one dose for three weeks, did not lead to any evidence of intestinal inflammation [91].

Accordingly, in a recent study, mice were fed with 5 μm pristine and fluorescent polystyrene MP for 6 weeks [93]. The results revealed that PS-MPs were observed in the intestine of mice, and reduced the intestinal mucus secretion, thus causing damage to the intestinal barrier function. Similarly, male mice exposed to polystyrene MP from 0.5 and 50 μm at 1000 μg/L for 5 weeks exhibited decreased intestinal mucus secretion following oral exposure [94]. On the other hand, when mice were exposed to different amounts of polyethylene microplastics, it led to histological inflammation in their colon and duodenum. Specifically, exposure to PE-MP (10–150 μm) at various concentrations (2, 20, and 200 μg/g for 5 weeks) resulted in the increased secretion of proinflammatory cytokines and higher levels of toll-like receptor 4 (TLR4), c-Jun, and interferon regulatory factor 5 [95].

Virgin PE spheres with a size between 45 and 53 μm and a concentration of 0.2 g/L (1.5 × 10^5^ particles/L) after 30 days of exposure have been found to cause impaired intestinal permeability in mouse models [96]. Another study on mammals confirms that exposure to MNPs may cause adverse effects on the intestinal system. When exposed to PS-NPs and PS-MPs (50 nm, 500 nm, and 5000 nm at a concentration of 20 mL/kg body weight for 28 days), there was a combined exposure that caused intestinal barrier dysfunction by the apoptosis of epithelial cells through ROS production in the mouse model [97]

In terms of toxicity, a mention must be made of the ability of MPs to transport pollutants and plasticizers. In this review, considering the focus of this paper; we will only refer to pollutants and plasticizers having the greatest potential to harm the gut system.

Chemical compounds called plasticizers can expose humans through occupational exposure, product use, or transfer from plastic packaging [33,107]. Exposure to these compounds can occur through ingestion, inhalation, and skin contact [108]. Among the various plasticizers, phthalates (PAEs) are known to be harmful to human health according to several studies. This group of chemicals is a major concern as they have been identified as endocrine-metabolic disruptors, which can affect the reproductive system based on available evidence from human epidemiological studies [109]. Numerous reports have found high levels of phthalate contamination in drinking water and various foods, including meat, oil, fats, dairy products, and even infant formula [110,111,112]. This suggests that these substances can easily enter the food chain, and ingestion may be the primary route of exposure [113,114]. Research has shown that the ingestion of various PAEs can lead to different health problems, such as reproductive, hepatic, cardiac, and neurodevelopmental disorders [115,116,117,118].

Although little research exists on how phthalates directly affect the intestinal system, these harmful substances are commonly found in contaminated food and water, making it highly likely that they negatively impact the gastrointestinal tract and gut microbiota.

Exposure of female CD-1 mice to phthalates at doses ranging from 0.2 to 200 mg/kg for 10–14 days caused colonic damage and inflammation. This was due to the dysregulation of the tight junction gene (Zo-3), cell cycle regulatory gene (Ccnb1), and cytokine levels (sICAM-1 and TNF-α) [119]. Additionally, Xiong et al. (2020) and Fu et al. (2021) observed elevated serum lipopolysaccharides (LPS) levels in mice exposed to PAEs, indicating epithelial barrier disruption and intestinal permeability [117,120]. Similarly, Deng et al. (2020) also reported reduced serum diaminoxidase (DAO) activity in CD-1 mice exposed to PAEs, which is an important indicator of impaired intestinal function [96]. Lastly, recent evidence suggests that the gut microbiota, due to its complex interaction with the intestinal epithelium and barrier, may play a significant role in influencing the local and systemic toxicity of these molecules [121]. 

It is widely acknowledged that PAEs and other plasticizers, including Bisphenol A, have the potential to negatively impact human health. In light of this, the European Food Safety Authority (EFSA) has recently advised lowering the acceptable daily intake (TDI) of such substances to safer levels [122,123].

Micro- and nanoplastics can also act as vectors for toxic heavy metals or other pollutants that can be released [124] into the environment and lead to health risks [38].

One example is chromium (Cr), which has a greater potential to adsorb on microplastics than other heavy metals. Microplastics can carry 19–7970 ng of Cr per g of microplastics [124]. When Cr (IV) enters the body, it causes DNA damage in various tissues at high acute doses or with chronic oral exposure [125]. To study the effect of the ingestion of adsorbed Cr on microplastics, ~150 μ PE, PP, PVC, and PS-MP contaminated with Cr at concentrations commensurate with water Cr-MP levels were prepared [124]. Using an in vitro method to model the entire digestive system, the researchers found that Cr (IV) availability was high for PLA in the stomach, small intestine, and large intestine. However, the risk quotients for adults and children calculated from the bioavailability did not raise concerns about the carcinogenicity.

### 3.2. MNP Gut Immunological Impact

The immune system present in the intestine is continuously exposed to external antigens, which are derived from food and non-pathogenic micro-organisms that need to be tolerated immunologically. However, the intestinal immune system also needs to be prepared to respond to pathogenic micro-organisms and external toxins. This balance is maintained by the equilibrium between pro- and anti-inflammatory stimuli, which involves innate lymphocytes, myeloid cells, and T- and B-lymphocytes residing in the lamina propria of the gut epithelium and draining in the mesenteric lymph nodes [126].

After being exposed to MNPs, immune cells trigger a significant modulation at the transcriptional level, affecting enzyme levels and cytokine release. Several studies, both on invertebrates and vertebrates, revealed an immune-toxic effect caused by nano- and microplastics on the intestinal immune system.

Exposure to PS nanoparticles has been found to cause higher hemocyte counts in *Daphnia magna*, while also decreasing the total antioxidant capacity and increasing DNA damage in mussels [127,128]. Amino-modified PS nanoparticles, on the other hand, have been shown to induce hemocyte changes in mussels, depending on the duration of exposure [129,130]. Additionally, exposure to PS microbeads or nanoparticles has been found to increase the production of oxygen reactive and nitrogen species, result in higher hemocyte mortality, and modify several enzymes related to the immune system, such as acid phosphatase, alkaline phosphatase, lysozyme, and phenoloxidase, depending on the duration and dose of exposure [131,132,133,134,135]. Studies have also shown that PS nanoparticles cause more damage than PS microparticles [134,135].

Studies on vertebrates have revealed some interesting findings. Exposure to PS nanoparticles led to a dose-dependent increase in myeloperoxidase activity and the release of neutrophil extracellular traps in fathead minnows *Pimephales promelas*. Similarly, polycarbonate microplastics dose-dependently disrupted neutrophil functions [136]. Exposure to PE microparticles in carp impaired the activity of the complement system and immunity-related enzymes [3]. Furthermore, in zebrafish, exposure to PE and PS particles reduced the liver transcript levels of two immune genes, leukotriene B4 receptor (ltb4r) and interferon-induced transmembrane protein (ifitm1) [86]. Furthermore, microplastics in the gastrointestinal tract have been found to upregulate the expression of T-cell receptors β and δ (TCRβ and TCRδ) and IgM in the spleen of *Scyliorhinus canicula* [137].

Lehner et al. (2020) developed a 3D in vitro intestinal model comprising human intestinal epithelial cell lines Caco-2 and HT29-MTX-E12 to study the effects of ingested MPs such as the 50-MP polymer of 500 μm representing tire wear and polyolefins at the concentration of 823.5–1380.0 μg/cm^2^. Although the results showed some changes in the levels of inflammatory cytokines (IL-8, TNFα, and IL-1β) and barrier integrity, these changes were not significant [99]. In contrast, other forms of MPs, polypropylene MPs (50–500 µm), have been shown to induce immune responses by triggering the production of proinflammatory cytokines such as IL-6 and TNF α in a size- and concentration-dependent manner [100].

A study on mice models has shown that exposure to PE microparticles can cause changes in the levels of certain proteins such as IL1α and granulocyte colony-stimulating factor (G-CSF) in the blood, a decrease in the count of regulatory T-lymphocytes, and an increase in the proportion of Th17 cells in the spleen [95]. In this study, it was found that high concentrations (600 μg/day) of PE-MPs (10–150 μm) caused inflammatory reactions by increasing the expression of Toll-like receptor 4 (TLR4), Activator Protein 1 (AP-1), and Interferon regulatory factor 5 (IRF5). The exposure to MP also led to a significant increase in the serum level of IL-1α and a decrease in Th17 and Treg cells in CD4+ T cells [95]. Additionally, PE microplastic exposure (40–48 μm per dosing volume of 200 μL/day for 90 days) can lead to an increase in the number of blood neutrophils and immunoglobulin IgA levels in female mice and an alteration of spleen lymphocytes in both dams and offspring [101].

While there is evidence of the effects of MNPs on the immune system, most studies have focused solely on the innate immune response, and the impact of MNPs on the adaptive immune response remains unclear.

A recent study [138] found that there is a connection between microplastics (MPs) in feces and inflammatory bowel disease (IBD). The study discovered that the fecal concentration of MPs in IBD patients was significantly higher (41.8 items/g dm) than in healthy individuals (28.0 items/g dm), including 15 different types of MPs. Among the MPs found, polyethylene terephthalate (22.3–34.0%) and polyamide (8.9–12.4%) were the most dominant types. The researchers observed that the primary shapes of the detected MPs were sheets and fibers [138]. Additionally, the study showed that there is a positive correlation between the concentration of MPs and the activity level of IBD, suggesting that MP exposure may be related to the disease process, or that IBD could promote the retention of MPs.

Indeed, further recent evidence in mouse models confirms these suspicions. It was observed that PS-NPs aggravate inflammation and intestinal injury in mice with chronic colitis [102]. Specifically, mice subjected to sodium dextran sulfate (DSS) exposures were subsequently fed via gastric tube with water containing 100 nm polystyrene nanospheres (PS-NPs, at concentrations of 1 mg/kg, 5 mg/kg, and 25 mg/kg) for 28 consecutive days. The results showed that PS-NPs exacerbated intestinal inflammation by activating the MAPK signaling pathway and also aggravated inflammation and oxidative stress in mice with chronic colitis.

These findings show that the intestinal immune system is altered by exposure to microplastics; however, further studies, especially in species more closely related to humans, are warranted.

### 3.3. MNP Effects on Gut Microbiota

The human gut is home to numerous communities of micro-organisms, collectively referred to as the “gut microbiota”. This microbiota comprises over 250 species of viruses, fungi, bacteria, and archaea, and is a dynamic system that changes over the course of a human’s life. The relationship between the gut microbiota and the host is mutually beneficial, as the former plays a crucial role in several physiological and pathological pathways of human life [139]. Human gut microbiota is primarily composed of five bacterial phyla: Firmicutes (60% to 80%), Bacteroidetes (20% to 40%), Verrucomicrobia, Actinobacteria, and a lesser extent of Proteobacteria; and one Archea phyla, the Euryarchaeota [139]. The gut microbiota is a crucial component of the gut ecosystem that plays a vital role in human health. It helps in the formation and maturation of immunity, acts as a barrier against pathogens, facilitates the absorption of nutrients and drugs, and regulates metabolic intake [140]. When there is an imbalance in the gut microbiota, it can lead to various gastrointestinal and extraintestinal disorders [140]. As a result, several therapeutic approaches, such as fecal microbiota transplantation [141], are increasingly being investigated for the treatment of microbiome-based disorders.

There is a lack of data concerning the effects of MNPs on the gut microbiota in humans. However, studies conducted on mammals have shown that both short- and long-term exposure to MNPs can cause modifications in microbial communities. Dysbiosis, or an imbalance in the gut microbiota, is a common finding in murine mole studies, with reduced alpha- and beta-diversity, and a loss of resilience. This can lead to frequent outbreaks of pathogens and metabolic disorders, both locally and systemically [93,95]. Particularly, at the phylum level, exposure to PS particles caused changes in the abundance of Bacteroidetes, Firmicutes, Actinobacteria, and Proteobacteria. At the genus level, variations in the abundance of *Staphylococcus*, *Clostridium*, and *Bacteroides* were observed when compared to animals that were not exposed to PS particles. In addition, up to 15 types of bacteria were affected by exposure to MPs, in particular, *Bifidobacterium*, *Prevotella*, *Veillonella*, Actinobacteria, and *Ruminococcus*.

Discrepancies were found regarding the abundance of Proteobacteria. In 2019, Lu et al. reported a decrease or increase in its abundance, while Jin et al. discovered a relative reduction in the abundance of Proteobacteria after PS microparticle exposure [93,94].

Conversely, in the same year, Luo et al. (2019) discovered that the Actinobacteria abundance increased while the abundance of Proteobacteria and Firmicutes remained unchanged in mice that were exposed to PS-MPs (0.5 µm and 5 µm) at a concentration of 100 µg/L and 1000 µg/L during their gestation and lactation period [103].

On the other hand, in 2021, Jiang et al. (2021) noted, following a 33-day period of the ingestion of 0.1 mg/kg MPs (5 µm), a shift in the relative abundance of bacterial taxa in mice models [104]. Specifically, there was a significant increase in the presence of Proteobacteria, while *Bacteroides* and *Marvinbryantia* exhibited a marked decrease. Additionally, *Bifidobacterium* also exhibited an increase. Qiao et al. confirmed mice exposed to PS-MNPs (70 nm, 5 μm in diameter) at a concentration between 2 mg and 0.2 mg kg^−1^ for 28 days experienced an increase in the relative abundance of Proteobacteria and Verrucomicrobia, while the major short-chain fatty acid (SCFA)-producing genera decreased in abundance [105].

In a study conducted by Liu et al. in 2022 [106], female mice were exposed to polyethylene terephthalate (PET) microplastics (ranging from 2 μm to 631 μm) at a concentration of 500 mg/kg for a period of 28 days. The study reported a decrease in the abundance of Bacteroidetes and an increase in the abundance of Firmicutes, which was accompanied by an increase in the abundance of *Lactobacillus* and a decrease in the abundance of *Parabacteroides*.

Two different studies focusing on PE microplastic exposure in mice, respectively, found an increase in the abundance of Firmicutes and Melainabacteria phyla and *Staphylococcus* genera, with a decrease in the abundance of Bacteroidetes phylum and *Parabacteroides* genera [95], and an increase in the abundance of the Actinobacteria phylum and *Lactobacillus*, *Adlercreutzia*, *Butyricimonas*, and *Parabacteroides* genera [96].

It is important to note that exposure to MNPs has been shown to reduce the abundance of bacteria that are known to promote tight junction functions. This reduction may have additional indirect toxic effects due to the dysbiosis of the gut microbiota [105].

Although plastic particles are inert to biodegradation due to their hydrophobic nature, high molecular weight, and long polymer chain, some micro-organisms ingest these polymers and convert them into environmentally friendly carbon compounds [142,143,144].

Polymer biodegradation is a process that occurs due to micro-organisms present in three domains of life, namely, Bacteria, Archaea, and Eukarya. Among the different kingdoms, fungi and bacteria are the most vital players in biodegradation processes in natural environments. The effectiveness of micro-organisms in degrading a specific type of plastic depends on the environmental conditions and the plastic typologies [145,146]. *Arthrobacter*, *Bacillus*, *Micrococcus*, *Pseudomonas*, *Corynebacterium*, *Streptomyces*, and *Nocardia* are the most commonly studied bacteria for their ability to degrade various types of plastics [147,148,149]. Besides free-living micro-organisms in the environment, the gut microbiota is an important driver of MNPs degradation, with most of the attention focused on insects and their larvae [150]. Indeed, several studies showed that MPs biodegradation does not occur after antibiotic treatment in mealworms, thus suggesting a crucial role played by the gut microbiota [151].

However, little is known about the microbial degradation capacity in mammals, probably due to the lack of appropriate high-resolution analytical methods to quantify small MPs and NPs and chemical intermediates in animal and human feces. Similarly, research regarding the microbial degradation of MPs and the human gut microbiota is still scarce; however, numerous plastic-degrading bacteria described in insects or larvae are part of the core of human gut microbiota, particularly, several potentially pathogenic Proteobacteria, such as *Enterobacteriaceae*, *Enterococcaceae*, *Listeria*, *Pseudomonas*, and *Klebsiella*, but also *Lactococcus* [152,153].

## 4. Conclusions

Annually, the global production of plastic waste amounts to millions of tons, a considerable quantity which disintegrates and accumulates in the form of minute particles that pollute and disseminate throughout terrestrial environments. Ingestion is a prevalent means of exposure of animals and humans to micro- and nanoplastics that can accumulate in the intestinal system to a degree and in a manner that remain incompletely understood.

Studies conducted in vitro on human cell lines have shown conflicting results regarding the toxicity of MNPs on the intestinal system. The discrepancies could be due to the different dosages of particles used in each study. Additionally, the various treatment periods and particle concentrations employed could also contribute to the conflicting nature of the findings. Furthermore, the studies cited only assess the short-term effects of MNPs on different endpoints, while possible long-term effects remain unexplored.

In contrast, studies conducted on mammals suggest that MNPs may have adverse effects in terms of intestinal cells toxicity, immunotoxicity, and dysbiosis. Nonetheless, the use of various study designs generates a degree of unclearness, and the absence of a definitive classification system for plastic waste based on parameters such as size, shape, and physical and chemical properties further complicates the issue. Additionally, the toxicological studies cited in this context do not account for the impact of realistic environmental exposure, nor do they consider the possible interactions between plastics and other pollutants.

Thus, we understand how these studies are not yet robust enough to determine their intestinal toxicity on mammals and humans with any degree of certainty. To gain a better understanding of the impact of MNPs ingestion on human gut health, it is essential to introduce validated and shared analytical methods. These arrangements will allow animal and cell studies to understand toxicological effects and will allow reference values to be generated to assess dietary intake and help stratify dietary risk. Observational and biomarker-based studies, on the other hand, will be able to help us unravel the real adverse effects of these particles on human gut health.

In conclusion, further studies and analytical methodologies are needed to characterize the real toxicological effects of MPNs on the intestinal human system and the precise role of the gut microbiota as a potential key player in this context.

## Figures and Tables

**Table 1:** Major studies investigating the potential effects of MNPs on the intestinal system in human cell lines and mammalian models.

Molecules	Species	Design	Size/Exposure	Effects	Study
Nanoplastics	Human	Human colon adenocarcinoma Caco-2 cell; in vitro design	PS particles between 50 and 200 nm at a concentration of 250 mg/mL for 10 to 120 min	Absence of cellular toxicity.	Abdelkhaliq 2018 [87]
Nanoplastics	Human	Human colon adenocarcinoma Caco-2 cell; in vitro design	100 nm PE terephthalate particles at a concentration between 1 and 30 mg/mL for an incubation time of 24 h	No evidence of increased inflammatory factors.	Magrì 2018 [88]
Nanoplastics, microplastics	Human	Human Caco-2 and HT29-MTX-E12 cells; in vitro design	50 nm and 0.5 μm COOH-modified PS particles, concentration (0.01 μg/mL–100 μg/mL) for an incubation time of 24 h	Absence of cellular toxicity.	Hesler 2019 [89]
Microplastics	Human	Human Caco-2 cells and gut microbiota; in vitro design	PE microplastics between 30 and 140 μm tested at various concentrations for 48 h	Significant reduction in Caco-2 cell viability, only for high concentrations (1000 mg/L).	Huang 2021 [90]
Microplastics	Human, mice	- Human colon adenocarcinoma Caco-2 cell; in vitro design - Male reporter gene mice; in vivo design	- 4 μm and 10 μm PS particles, variable concentration for an incubation time of 48 h - Mixture of 1 µm to 10 µm PS microplastics at a volume of 10 mL/kg and a total of one dose for 3 weeks	- Reduction in cell vitality for high concentrations (1 × 10^8^ particles/mL); no effect on cell polarization. - Absence of histologically detectable lesions and inflammatory responses.	Stock 2019 [91]
Microplastics	Human	HRT-18 and CMT-93 epithelial human cell lines; in vitro design	PS microparticles of 4.8–5.8 µm for a concentration of 1 mg/mL and a time between 6 and 48 h	Significant cytotoxicity in both cell lines. Oxidative stress activity was increased only in CMT-93 cells.	Mattioda 2023 [92]
Microplastics	Mice	IRC mice divided into control and exposed group; in vivo design	PS microparticles of 5 µm for a concentration of 100 and 1000 µg/L for six weeks	Reduced mucus production and damage to the intestinal barrier. Decreased Actinobacteria content and altered microbial alpha diversity. At the genus level, a total of 15 types of bacteria changed significantly.	Jin 2019 [93]
Microplastics	Mice	Male mice exposed to two different MP sizes; in vivo design	Oral exposure to 1000 μg/L of 0.5 and 50 μm PS-MP for five weeks	Decreased mucus secretion in the intestine in both sizes of treated groups. Decreased relative abundance of Firmicutes and α-Proteobacteria in the feces. Significant changes in the richness and diversity of the caecal intestinal microbiota.	Lu 2018 [94]
Microplastics	Mice	SPF grade C57BL/6 male mice were divided into four groups; in vivo design	Exposure to different amounts of PE microplastics between 10 and 150 μm (6, 60, and 600 μg/day for 5 consecutive weeks)	Induction of histologic inflammation in the colon and duodenum (a higher expression of TLR4, AP-1, and IRF5). Changes of IL1α and granulocyte colony-stimulating factor (G-CSF) in the blood, decrease in the count of regulatory T-lymphocytes, and an increase in the proportion of Th17 cells in the spleen. Increased number of intestinal microbial species, bacterial abundance, and diversity of flora. Significant increase in *Staphylococcus* abundance along with a significant decrease in *Parabacteroides* abundance.	Li 2020 [95]
Microplastics, phthalate esters	Mice	Male mice (Mus musculus CD-1) divided into 12 groups and exposed to MPs and MPs contaminated with phthalate esters; in vivo design	Virgin PE spheres of size between 45 and 53 μm and concentration of 0.2 g/L (about 1.5 × 10^5^ particles/L) for 30 days of exposure	Disruption of intestinal permeability. Increased abundance of phylum Actinobacteria and genera *Lactobacillus*, *Adlercreutzia*, *Butyricimonas*, and *Parabacteroides*.	Deng 2020 [96]
Microplastics, nanoplastics	Mice	6-week-old C57BL/6 J mice; in vivo design	Combined exposure to PS-NP and PS-MP (50 nm, 500, and 5000 nm, respectively, at a concentration of 20 mL/kg body weight for 28 days)	Gut barrier dysfunction by apoptosis of epithelial cells through ROS production.	Liang 2021 [97]
Microplastics, nanoplastics	Human, mice	- CCD18-Co cells from normal human colon fibroblasts, human colon organoids; in vitro design - Seven-week-old male C57BL/6 mice; in vivo design	- Exposure to 50- and 100 nm MNP particles at varying concentrations for 48 h of incubation - 50 nm MNPs at the concentration at which the highest toxicity was found in colonic organoids, for 7 days	Concentrations of 5 mg/mL induced > 20% decrease in colonic organoid viability and increased expression of genes related to inflammation, apoptosis, and immunity. 50 nm MNPs accumulate in various mouse organs, including colon, liver, pancreas, and testes after 7 days of exposure.	Park 2023 [98]
Microplastics	Human	3D in vitro intestinal model comprising human intestinal epithelial cell lines Caco-2 and HT29-MTX-E12	Exposure to 50–500 µm MP at the concentration of 823.5–1380.0 μg/cm^2^ for 24 h	No induction of cytotoxicity, nor pro-inflammatory response.	Lehner 2020 [99]
Microplastics	Human, mice/sheep	Murine and sheep blood and immune cells; human-derived cell lines; in vitro design	Polypropylene MPs (50–500 µm) at various concentrations	Induction of proinflammatory cytokines in a size- and concentration-dependent manner.	Hwang 2019 [100]
Microplastics	Mice	Six-week-old male and female ICR mice; in vivo design	40−48 μm PE-MPs (0.125, 0.5, 2 mg/day/mouse) by gavage to mice (10 mice/sex/dose) for 90 days	Increase in the number of blood neutrophils and immunoglobulin IgA levels, alteration of spleen lymphocytes.	Park 2020 [101]
Nanoplastics	Mice	Mice with chronic colitis; in vivo design	100 nm polystyrene nanospheres (PS-NPs, at concentrations of 1 mg/kg, 5 mg/kg, and 25 mg/kg) for 28 consecutive days	Increase in oxidative stress and intestinal inflammation by activating the MAPK signaling pathway.	Ma 2023 [102]
Microplastics	Mice	Male and female ICR mice; in vivo design	PS-MPs (0.5 µm and 5 µm) at a concentration of 100 µg/L and 1000 µg/L, from day 1 of gestation to the day of birth	Abundance of Actinobacteria increased, while that of Proteobacteria and Firmicutes remained unchanged.	Luo 2019 [103]
Microplastics	Mice	Seven-week-old male C57BL/6J mice; in vivo design	Oral exposure of 5 µm MPs (0.1 mg/day) for 33 days	Increased relative abundance of Proteobacteria. Decrease in *Bacteroides* and *Marvinbryantia* and increase in *Bifidobacterium*.	Jiang 2021 [104]
Microplastics, nanoplastics	Mice	C57/B6 mice (male, 8 weeks old); in vivo design	PS M/NPLs, and carboxyl-modified (PS-COOH) and aminomodified (PS-NH2) PS M/NPLs (70 nm, 5 μm in diameter), at a concentration between 2 mg and 0.2 mg/kg, for 28 days	Increased relative abundance of Proteobacteria. Increase of Verrucomicrobia at a high concentration. Reduced several short-chain fatty acid (SCFA)-producing genera.	Qiao 2021 [105]
Microplastics	Mice	4-week-old female mice (KM mice); in vivo design	PET-MPs (2 μm to 631 μm) at a concentration of 500 mg/kg for 28 days	Decreased abundances of Bacteroidetes and increased abundance of Firmicutes. Increased abundance of *Lactobacillus* and decreased abundance of *Parabacteroides*.	Liu 2022 [106]

MP: microplastic; NP: nanoplastic; MNPs: micro- and nanoplastics; PS: polystyrene; PE: polyethylene; PET: polyethylene terephthalate.

## Data Availability

Not applicable.
